# Validation of “Self-Evaluation of Communication Experiences after Laryngectomy” (SECEL) Questionnaire for Spanish-Speaking Laryngectomized Patients

**DOI:** 10.3390/cancers14143347

**Published:** 2022-07-09

**Authors:** Eva Villanueva, María Paula Fernández, Giovanna Arena, José L. Llorente, Juan P. Rodrigo, Fernando López, César Álvarez-Marcos

**Affiliations:** 1 Department of Otorhinolaryngology and Head and Neck Surgery, Hospital Universitario Central de Asturias (HUCA), 33011 Oviedo, Spain; evaviva12@hotmail.com (E.V.); jllorentep@uniovi.es (J.L.L.); juanpablo.rodrigo@sespa.es (J.P.R.); alvarezmarcos@telefonica.net (C.Á.-M.); 2 Faculty of Psychology and Speech Therapy, University of Oviedo, 33003 Oviedo, Spain; paula@uniovi.es (M.P.F.); giovannaagl@gmail.com (G.A.); 3 Instituto de Investigación Sanitaria del Principado de Asturias (ISPA), Instituto Universitario de Oncología del Principado de Asturias (IUOPA), University of Oviedo, IUOPA, CIBERONC-ISCIII, 33011 Oviedo, Spain

**Keywords:** total laryngectomy, communication, voice, quality of life, SECEL

## Abstract

**Simple Summary:**

There is no tool in Spanish that assesses communication-related quality of life in laryngectomized patients. Therefore, we have translated and adapted the SECEL scale from English to Spanish and applied it to a group of patients to test its reliability and validity. As a result, we have observed that the Spanish version of the SECEL questionnaire has the same conceptual, semantic and idiomatic meaning as the original. The resulting model is composed of 21 items that can be used in clinical practice in a reliable way. The test can be considered a suitable tool to assess the communication skills of Spanish-speaking laryngectomees.

**Abstract:**

Background: Laryngectomized patients have communicative limitations when they lose their phonatory function after surgery. The scale “Self-Evaluation of Communication Experiences after Laryngectomy” (SECEL) assesses the impact of total laryngectomy on quality of life related to communication. The aim of this study was to translate and adapt the SECEL from English to Spanish and to apply this first version to a group of patients to check its reliability and validity. Materials and Methods: One-hundred-and-one laryngectomized patients completed the SECEL scale adjusted to Spanish, the Voice Handicap Index (VHI-30), the Hospital Anxiety and Depression Scale (HADS) and the EORTC QLQ-H&N35 questionnaire. Results: The Spanish version of the SECEL questionnaire has the same format and conceptual, semantic and idiomatic meaning as the original. The resulting model is composed of 21 items dimensioned in two highly correlated factors that are clear, meaningful, and replicable leading to measures that are reliable enough to be used in clinical evaluation. Conclusion: The Spanish translation and validation of the SECEL questionnaire were performed. It can be considered an appropriate tool to assess communication skills from laryngectomized people.

## 1. Introduction

Total laryngectomy (TL) causes an important disruption in respiratory, olfactory, deglutory and phonatory functions [1,2]. All these changes are related to a significant impairment in patients’ social and personal aspects, limiting their social interactions and increasing their symptoms of anxiety and depression [3].

The loss of phonatory function is the most important change that takes place after a TL [4]. The impact on communication and adaptation to a new reality following a TL has been described. It has also been observed that there is a strong association between communication skills and quality of life. (QoL) [3,5]. For this reason, vocal rehabilitation is essential to reduce their feelings of loneliness and avoid potential psychosocial issues [6,7].

Being aware of the needs, expectations, and capabilities of laryngectomized patients is essential to indicate an appropriate rehabilitation therapy in order to achieve a rehabilitation program as personalized as possible. There are several questionnaires regarding the QoL of these patients, but none of them give due importance to communication aspects. The European Organization for Research and Treatment of Cancer (EORTC) [8] questionnaire in its specific version for head and neck cancer (QLQ-H&N35) has only four questions related to voice disorders and communication. Other questionnaires designed for the detection of voice impairment, such as VHI-30 (Voice Handicap Index) [9] or CAPE-V (Consensus Auditory Perceptual Evaluation) [10], are not adapted to people with an alternative method of communication to laryngeal voice.

The SECEL (Self-Evaluation of Communication Experiences after Laryngectomy) questionnaire [11] is specifically designed to evaluate the communication skills of laryngectomized patients. It contains 35 specific questions, split into three scales: general, environment and attitude. This tool has proven to be more sensitive than other questionnaires assessing communication disorders [12]. Therefore, it could be an excellent instrument to develop recommendations to achieve personalized voice therapy and rehabilitation.

SECEL has already been translated into five languages: Swedish [13], Italian [14], European Portuguese [15], Brazilian Portuguese [16], and Turkish [17]. However, there are a lack of specific instruments applied to assess communication problems after TL in Spanish. Spanish is the second most widely spoken language in the world, with approximately 600 million native speakers, and with official status in 21 countries. Therefore, the translation and adaptation of this questionnaire into Spanish would benefit a significant number of laryngectomized patients.

The aim of this study was to translate, adapt, and validate the Spanish version of the SECEL in a series of patients with a TL. This adaptation is intended to provide a better instrument to analyze the QoL of Spanish-speaking laryngectomized patients, paying attention to communication problems and facilitating the rehabilitation process.

## 2. Materials and Methods

### 2.1. Design and Participants

We developed a cross-sectional study at the Otorhinolaryngology Department of the Hospital Universitario Central de Asturias (HUCA) (Asturias, Spain) and the AECC (Asociación Española Contra el Cáncer) from January 2020 to January 2021. All procedures were conducted in accordance with the Declaration of Helsinki and approved by Institutional Ethics Committee of the HUCA (118/19). Written informed consent was obtained from each patient. We included 101 patients who had undergone a primary TL for laryngeal cancer. All of them had completed their speech rehabilitation process. The inclusion criteria included patients with head and neck cancer who spoke Spanish as their native language, treated with a TL and a time interval since surgery of at least 9 months. Those patients who presented physical or cognitive impairment or could not correctly complete all the questionnaires for another reason were excluded. A descriptive study of patients’ demographic characteristics and clinical features was detailed.

As these were self-administered questionnaires, the presence of the researcher was not essential to the completion process. Eighty-eight patients (88.8%) were interviewed at the time of their follow-up visit. Each participant and their companions had the proper way to conduct the questionnaires explained to them during their completion in person. The other 13 patients (13.1%) were mailed the questionnaires due to their review being postponed because of the COVID-19 pandemic. In this case, an instruction sheet was included, and telephone support was also provided when necessary. Throughout the procedure, different questionnaires were presented randomly to avoid order effects.

All but five patients completed again the SECEL questionnaire 4 weeks after the first (retest), without access to their previous answers, to check the stability of patients’ answers to the first application of the questionnaire.

### 2.2. Translation and Cultural Adaptation

In the translation and cultural adaptation of the SECEL questionnaire into Spanish, backtranslation was used, which is a widely used method for quality control in translation and is considered to be the most comprehensive method [18,19,20]. The original version of the SECEL questionnaire was translated from English into Spanish by two bilingual translators whose native language was Spanish, independently of each other. Both versions (T1 and T2) were combined into a common version (T12) which was administered to five patients in a pilot study, asking about difficulties in understanding and formatting of the questionnaire. We then developed the latest version of the T12 with modifications based on patient feedback. This version was then back-translated into the original language by two bilingual translators whose native language was English to verify the validity of the content, resulting in versions BT1 and BT2. Finally, translators and researchers discussed discrepancies and differences between all these versions, and we developed the initial version of the SECEL scale in Spanish (S-SECEL).

### 2.3. Measuring Instruments and Questionnaires

The original SECEL [11] is a self-administered questionnaire with 35 items divided into 3 subscales (general, attitude and environment) aimed at assessing the communication skills of laryngectomized patients. The first 34 items are to be answered on a four-point scale (0: never, 1: sometimes, 2: often, 3: always) and the last item asks the patient whether he/she talks the same, more or less than before surgery and why. This questionnaire provides information about the perceived difficulty with their new method of communication. A total score is obtained with a range of 0–102 points; higher scores, especially >60 points, indicate a higher perceived difficulty with the adaptation to the new method of communication and suggest the need for further rehabilitation.

For the assessment of validity, patients completed three other questionnaires, as carried out in the validation process of SECEL to other languages [13,14,15]. These questionnaires validated in Spanish are:

- QLQ-H&N35 [8]: a self-administered questionnaire developed by EORTC to assess the perceived QoL in patients with head and neck cancer. It consists of 7 subscales with 35 items. Each item has a four-point scale (1: not at all, 2: a little bit 3: pretty much, 4: a lot). The scale score is transformed into a 0-to-100 scale and a high score on a symptom scale indicates a high symptom level. In this study, those patients who exceed the 75th percentile score are considered to have a poor QoL.
- VHI-30 [9]: a self-administered questionnaire with 30 items divided into 3 subscales (Emotional, Functional and Physical) to assess voice disorders. Each item is scored from 0 to 5 (0: never, 1: hardly ever, 2: sometimes, 3: often, 4: always). According to the total score, four degrees of voice handicap are established, ranging from mild (0 to 30), moderate (31 to 60), severe (61 to 90) and grave (91 to 120).
- HADS [21]: a self-administered mental health questionnaire developed for the detection and evaluation of mood disorders. It has 14 items divided into 2 subscales: Anxiety (HAD-A) and Depression (HAD-D). Patients rate each item on a 4-point scale (0–3), choosing the most representative number of their situation the week before. Scores > 11 in either of the subscales indicate probable psychological distress. Scores > 10 are considered indicative of morbidity. A score of 8–10 is interpreted as borderline or borderline case, and scores < 8 indicate the absence of significant morbidity.

### 2.4. Data Analysis

The sample, despite consisting of only 101 participants, is considered to be representative of the population it is intended to represent. These data are important for the validation of a questionnaire. This same assumption was considered by Blood (1993) in the process of constructing the SECEL scale, and also by all researchers who have translated and adapted the scale into languages other than the original language, due to the difficulty involved in having a large sample size in this particular case. The physical change and the difficulties that laryngectomized people experience in their lives are very particular and well-defined. However, in the process of validating a questionnaire, in addition to the sample being representative, the sample must be of sufficient size for the statistical analyses to be reliable and stable. For this reason, in the steps carried out in the validation process, precautions have been taken to guarantee both aspects, which are described in this section. Thus, in detail, the data analysis was carried out as described below.

First, the dimensionality of the 34-item, 3-factor model proposed by Blood [11] was tested with Confirmatory Factor Analysis (CFA) and Semi-Confirmatory Factor Analysis (sCFA) [22,23,24]. This step is necessary when starting a validation study [24]. As the number of items is high in relation to the sample size, it was considered necessary to perform this assessment in both ways, and to consider the result valid if both analyses converge. Both methods concluded that the Blood model (M0) did not fit the data obtained in Spanish participants. This could be due to having a sample size that is not very large, but as the test results are convergent, we assume that the results are true.

Therefore, its dimensionality was adjusted using an exploratory factor analysis (EFA), which is a commonly used method to determine the dimensionality of continuous data. For the same reason as stated above concerning the sample size in relation to the number of items, it was decided before starting the EFA to eliminate all items that were found to be unsuitable for the questionnaire as a result of the descriptive study. Experts advise eliminating items that are not very discriminative (items whose mean is close to the highest or lowest value of the Likert scale used, items whose standard deviation is less than 1, and items whose asymmetry and/or kurtosis > 1.5 ([22,23]). On the other hand, experts advise eliminating items whose content is not sufficiently related to the content of the scale (items with a corrected homogeneity index (HIc) < 0.30) [24]. Based on both aspects, 9 items were removed before starting the EFA (this aspect is detailed in the results section). Next, in successive EFAs, items with a factorial load < 0.40 and complex items were also eliminated (there were 4 items in total), making the pertinent adjustments in each successive model, until a simple and interpretable final model was obtained [23]. Finally, the 21-item model M1 was tested using the CFA.

The sCFA was performed using the FACTOR software (V.11.04.02) (Rovira i Virgili University, Tarragona, Spain). FACTOR uses procrustean rotations against a target matrix and examines the fit of models based on the Root Mean Square Deviation (RMSD). If RMSD < 0.05, the misfit is trivial; between 0.05 and 0.10, it is moderate; and if greater than 0.10, the misfit is substantial. The EFA was also performed using FACTOR, and the CFA was performed using JASP v0.14.1. https://jasp-stats.org/download/ (accessed on 10 December 2020) [22,23,24,25]. The estimation procedure for all EFAs was the unweighted least squares (ULS), determining the number of factors through the optimal implementation of Parallel Analysis, using the Oblimin rotation [26].

The adequacy of the dimensionality of the models was checked using the Kaiser–Meyer–Olkin (KMO) index, Bartlett’s sphericity test, the Bayesian Information Criterion (BIC), and the Comparative Fit Index (CFI) [22,24,27,28]. Residual values were evaluated by the Root Mean Square Error of Approximation (RMSEA), Root Mean Square of Residuals indices (RMSR), Standardized Root Mean Square of Residuals (SRMR) and the χ2/df ratio [22,24,29]. The evaluation of the simplicity of both the model and the load of the items was carried out using the Bentler simplicity index (S) [30].

The closeness to unidimensionality was assessed using the UniCo and ECV (Explained Common Variance) indices, and the Value-added analyzes (Add-V) using prediction mean square error (PRMSE). Data can be treated as essentially one-dimensional when UniCo > 0.95 and ECV > 0.85 and if PRMSE is lower in the corresponding factors than in the general factor [31].

The internal consistency analysis was analyzed using Composite Reliability (CR) derived from the CFA result and considering the standardized factor loadings [32]. The reliability of the measurement of the resulting scale (S-SECEL) was estimated using McDonald’s ordinal omega and Cronbach’s standardized alpha [33]. Temporal stability (test–retest) was estimated with Pearson’s correlation (one month between both measurements).

Finally, the external convergent validity was studied using Pearson’s correlation between the S-SECEL factors and the metric variables of mental health, anxiety, and depression (HAD-A, HAD-D), QoL (QLQ-H&N35) and vocal disability (VHI-30) [34].

## 3. Results

### 3.1. Sample Characteristics

Most participants in the sample were men of Spanish nationality, married, with primary education and retired from work. More than half had received complementary treatment for their TL with radiation and/or chemotherapy. The most used communication method was the esophageal voice with a variable degree of intelligibility. At the time of data collection, the patients in the sample had a mean age of 68 years (range 43–88 years). The demographic characteristics and clinical features of the 101 patients are detailed in Table 1.

Regarding the variables referring to HADS, most patients did not have symptoms of anxiety or depression (81.2% and 82.8%, respectively) and 70.7% had no symptoms of any of them. The QLQ-H&N35 shows a good QoL since the maximum score achieved in the patient sample is 80, with the maximum possible being 140 (the higher the score, the worse QoL). Regarding vocal disability (VHI-30), the majority state that they have a moderate disability (57.4%) (Table 2).

### 3.2. Validity Evidence Based on the Internal Structure and Reliability of Scale Score

The translation of the SECEL questionnaire to the Spanish language was performed, and the complete version is shown in Table 3.

The results of the evaluation of the dimensionality and internal consistency of the SECEL questionnaire are shown in Table 4 and Table 5.

The model originally described by Blood [11] of 34 items and 3 factors, M0, does not fit the data from the sample of Spanish participants. Both sCFA and CFA methods conclude the same result. In the sCFA, the RMSD values were 0.112, 0.198, and 0.171 for the General, Environmental, and Attitudinal factors, respectively, indicating substantial mismatch, with a total mean mismatch of 0.162. The initial CFA showed unsatisfactory fit only in SRMR [χ2/df = 0.77; CFI = 1; SRMR = 0. 086 and RMSEA = 0] (Table 4), but the standardized factor loadings of items 2, 4, 15 and 29 were not statistically significant, and the loadings of items 3, 8, 31, 32 and 34 were below 0.30 (Table 5). The internal consistency of the General, Environmental and Attitudinal subscales identified by Blood was 0.208, 0.853 and 0.858, respectively (Table 5).

Before starting the EFA, nine items were eliminated: items 2, 3, 4, 8, 15 and 29 because they had a HIc < 0.30, and items 31, 32 and 34 because they have a mean close to the highest or lowest value of the scale (>2.40 or <0.60 in this case), have a SD less than 1, and also asymmetry and/or kurtosis> |1.5|). These nine items are unusable items. The first items are unusable because they do not correlate sufficiently with the content of the questionnaire as a whole, and the last three because they do not allow us to capture the variability between the subjects in the sample, with the addition that in the CFA of the theoretical model, M0, all of these items are either not statistically significant, or are statistically significant but have a standardized factor loading of less than 0.40 (Table 5). Thus, a modeling process was initiated through successive exploratory factor analyses (EFA) with 25 items. The adequacy of the data examined by means of the KMO sphericity test and Bartlett’s test was always good, and items 1, 11, 28 and 30 with factor loading < 0.40 were eliminated in successive modelling. In summary, after adjustment and the modeling process, a total of 13 items were eliminated. It was concluded that Model M1, sized with 2 factors consisting of a total of 21 items (F1 and F2, which have 12 and 9 items, respectively), is the simplest model and best adjusted one (Table 4 and Table 5). The percentage of variance explained by both factors is 54.93% (F1 explains 43.64% and F2 explains 11.29%). The corresponding autovalues were 9.16 and 2.37, respectively.

The UniCo = 0.851 and ECV = 0.982 indices show that the structure of the M1 model is far from unidimensional. This aspect was corroborated by the added-value analyses, since the PRMSE prediction made from the two factors found (0.930 and 0.918 for F1 and F2) was greater than that estimated from a general factor (0.735 and 0.455, respectively).

The CFA confirmed a satisfactory fit of Model M1 [χ2/df = 0.50; CFI = 1; RMSR/SRMR = 0.065 and RMSEA = 0], to a greater extent than the fit of the one-dimensional model M2 [χ2/df = 0.65; CFI = 1; RMSR/SRMR = 0.073 and RMSEA = 0] (Table 4), and also CR was high in F1 (0.916) and F2 (0.845) (Table 6). The internal consistency evaluated by Cronbach’s alpha, McDonald’s ordinal omega test and CR was very high, greater than 0.80 in both factors (F1 and F2) (Table 6). Finally, the test–retest correlation was satisfactory (r = 0.69; 0.79 and 0.74 for F1, F2 and total) in the M1 model (S-SECEL), indicating an excellent temporal stability.

### 3.3. Validity Evidence Based on the Relationship with Other Variables

Pearson’s correlations between the scores derived from the S-SECEL and the HADS (HAD-A, HAD-D), QLQ-H&N35 and VHI-30 are all positive and statistically significant (Table 7). In these mental health, QoL and perceived vocal disability questionnaires, the higher the score, the worse the patient experience. These results mean that the greater the vocal disability experienced by the patients (VHI-30), the worse the QoL (QLQ-H&N35) and the greater likelihood of the experience of depression and anxiety (HADS), with F1 being the most affected factor in the questionnaire (Table 7).

**Figure 1:**
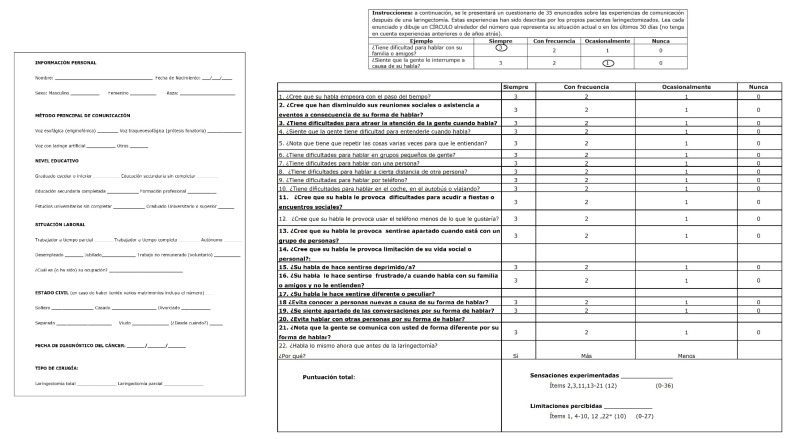
Self-Evaluation of Communication Experiences after Laryngectomy–Spanish adaptation (S-SECEL).

Once the factorial analysis was completed and the dimensionality, reliability and concurrent external validity were studied, the obtained results suggest that the best solution to apply SECEL in the Spanish population (final version of the S-SECEL) (Figure 1) was 21 items fitted into two moderately correlated factors (r = 0.636 and 0.702 in EFA and CFA, respectively), (Table 4). Factor 1 (F1) would be composed of 12 items: 6, 7, 18, 20, 21, 22, 23, 24, 25, 26, 27 and 33 (items highlighted in bold in Figure 1), while factor 2 (F2) would be composed of 9 items: 5, 9, 10, 12, 13, 14, 16, 17 and 19 of the original SECEL numbering 8 (items not highlighted in bold in Figure 1). We have called Factor 1 “Experienced sensations”, whose items are mostly part of what Blood [11] called the attitude subscale, and Factor 2 “Perceived Limitations”, whose items are mostly part of what Blood [11] called the subscale environment. Item 35 of the original SECEL scale, which is not numerically assessed, has been included in this last subscale.

## 4. Discussion

The validation and adaptation of the SECEL into Spanish would allow its use in the Spanish-speaking population, the language with the second largest spread worldwide at 600 million speakers, being the official language in 21 countries, some with very high rates of laryngeal cancer. Although the Spanish language is uniform, there may be spelling variations between different Spanish-speaking countries with different dialects or accents. However, as with other languages, the difference is not so prominent, and all Spanish speakers are able to understand the language. Knowing the communication skills of laryngectomized people will allow the establishment of reference points on which to carry out their vocal rehabilitation in a personalized way, attending to their vocal needs and their mental state [1,2].

The S-SECEL was developed according to the guidelines for the cross-cultural adaptation of PROs (Patient-Reported Outcomes) [18,19,20]. It is congruent with the original SECEL and the other translated versions in format, content and scoring system.

Regarding the translation, we did not observe differences between the original version and the Spanish version of the SECEL. Furthermore, in the back-translation, both translators agreed than item number 5 (Do you think your speech improves with the amount of time you use it?) and 14 (In different rooms of your house (apartment, residence)) were unclear in the Spanish version, so we decided to modify them similar to the Brazilian Portuguese model (16). In addition, in item 5, which was formulated in an inverse way, “mejora” (improves) was replaced by “empeora” (worsens), so that all the items assessed the communication problem directly and did not have to assign it an inverse value, taking as a reference what had been performed in other studies [35]. Items that used the word “habla” (speech) referring to patient’s new way of communication (items 1, 3–13 and 21) were also problematic, so they were modified to “forma de hablar”. This problem was also observed in the Portuguese version [16]. Finally, “¿por qué?” (why?) was added to item 35 with the aim of obtaining more information. This item does not add a numerical value to the score.

The study was carried out with 101 patients, a sample similar to those used in other validation studies of the original Blood questionnaire [11]. Patients’ characteristics are similar to the populations in other studies (the majority are married and retired men with a mean age between 43 and 88 years old). All the patients had a TL (Table 1). This homogeneity provides added value to the assessment of communication skills. Other validation studies include patients treated with exclusive radiotherapy or partial laryngectomies that preserve, to a greater or lesser extent, the ability to speak with a laryngeal voice [13,14,16]. One of the advantages of translating SECEL into several languages is that this will allow international cross-cultural comparison, contributing for the improvement of health care quality.

We estimate, therefore, that the sample is representative of the population it is intended to represent. However, given the small sample size in relation to the number of items in the original scale, all data analyses were carried out in order to optimize statistical resources and control of the research.

Our study shows that the best option for adapting the original SECEL to the Spanish-speaking population is a 21-item questionnaire fitting into two moderately correlated factors (r = 0.636 and 0.702 in EFA and CFA, respectively). Factor 1 is composed of 12 items: 6, 7, 18, 20, 21, 22, 23, 24, 25, 26, 27 and 33; and Factor 2 by 9 items: 5, 9, 10, 12, 13, 14, 16, 17 and 19 (original scale numbering). We have denominated Factor 1 “Experienced Sensations” and Factor 2 “Perceived Limitations”.

All the items of Factor 1 of the Spanish version of SECEL were part of the original scale [11] named “attitude”, except for items 6 and 7 which belonged to the “environment” scale. The items of Factor 2 of SECEL belonged to the “attitude” scale of the original test, except for item 5, which belonged to the “general” scale. These data have been corroborated in the adaptation of the SECEL to the Swedish language [13], although it does not specify them, suggesting that this overlap could be due to the fact that the content of these items was misunderstood by the participants due to ambiguous wording. “Experienced sensations” and “perceived limitations” of the Spanish version of the SECEL behave with the variables of mental health, QoL and vocal disability in a similar way as the “attitude” and “environment” scales of the original SECEL do, although with greater discrimination when relocating the items in the most related factor and by eliminating those that do not show variability.

In previous adaptations into other languages, it was observed that the internal consistency of the “attitude” and “environment” scales is high, but in the “general” scale it is very low [13,14,15]. This can be explained because the “general” scale has only five items that assess different aspects of laryngectomized patients, thus generating the error. The consequence is that the “general” scale contributes very little value to the instrument, conserving in the Spanish version of the SECEL only item 5 that was integrated in “perceived limitations”. The internal consistency of the two Spanish versions of the SECEL factors was very high and the test–retest correlation was adequate, with the temporal stability being greater in “perceived limitations” than in “experienced sensations” (Table 6). F1 (“experienced sensations”) is more unstable since it will depend on the state of mind and the emotional capacity to overcome the situation. It is likely that its alteration means that the patient, in addition to vocal rehabilitation, needs psychological support. F2 (“perceived limitations”) is more stable and would be related to the inability to communicate, although it could improve if the voice is rehabilitated. The values of these subscales could vary over time, improving or worsening depending on the personal circumstances of each patient or the therapies performed.

There are some limitations regarding the current study that should be mentioned and addressed in future studies. A relative limitation is the size of the sample, although it is larger than other validation studies in other languages and only has one less participant than the sample used by Blood to study the reliability and validity of the original questionnaire [11]. One aspect that we consider a strength is that all the participants had been treated with TL. These patients have greater communication problems with loss of voice, so the questionnaire would not be indicated in partial laryngeal surgery or other vocal disorders where only voice quality would be altered. However, a possible limitation of our study is the high rate of patients who use esophageal speech as a mode of communication compared to tracheoesophageal prothesis or artificial larynx, which could imply a lack of generalizability of the study. The results, although robust, are preliminary and have been obtained from a cross-sectional study; it is necessary to apply the questionnaire in the clinic to establish cut-off points and see its evolution after vocal rehabilitation or psychological therapy, as other authors have already pointed out [12,36]. A longitudinal study is currently being undertaken to observe the evolution of patients as they progress in their rehabilitation, with special emphasis being placed on planning the sample size to consider the loss of data, a common problem in this type of research [37,38]. All these limitations generate new perspectives for future studies, such as increasing the number of participants, establishing variations of the questionnaire for different laryngectomy modalities, or making comparisons between different countries.

## 5. Conclusions

The Spanish version of SECEL or S-SCECEL is a valid and reliable instrument, with a solid internal structure and strong convergent validity, which allows its use to assess communication problems in patients undergoing TL. The adaptation process of the original questionnaire obtained a reduced version of 21 items and 2 scales, which were clear, simple, significant, and replicable, facilitating its completion. In the clinic, it would allow for a better evaluation of patients with vocal communication dysfunction, in addition to assessing their state of mental health, to adapt, improve and personalize their vocal rehabilitation process.

## Figures and Tables

**Table 1:** Demographic characteristics and clinical features.

Demographic Characteristics	No. (%)
Age	68 (43–88)
Sex	
Man	92 (91.1)
Woman	9 (8.9)
Nationality	
Spanish	98 (97)
Foreign	9 (8.9)
Working condition	
Retired	89 (88.1)
Not retired	13 (11.9)
Marital status	
Married	70 (69.3)
Other	31 (30.7)
Education	
Primary	62 (61.2)
Secondary	25 (24.8)
University	14 (13.9)
Clinical features	
Adjuvant treatment after TL	
RT	37 (36.6)
CT	7 (6.9)
CRT	13 (12.9)
No	44 (43.6)
Type of voice	
Esophageal	80(79.2)
Tracheoesophageal prosthesis	4 (4)
Artificial larynx	7 (6.9)
Other	10 (9.9)
Perceptual intelligibility	
Does not speak	11 (10.9)
Monosyllabic	39 (38)
Faulty speech	21 (20.8)
Intelligibility speech	30 (29.7)

CR: chemo-radiotherapy; CT: chemotherapy; RT: radiotherapy; TL: total laryngectomy.

**Table 2:** Mental health, quality of life and vocal disability.

			No. (%)
**Mental health variables**	HAD-A (*n* = 101)	No	82 (81.2)
		Doubtful	10 (9.9)
		Probable	9 (8.9)
	HAD-D ^1^ (*n* = 99)	No	82 (82.8)
		Doubtful	9 (9.1)
		Probable	8 (8.1)
	HAD-A&D ^1^ (*n* = 99)	No anxiety and no depression	70 (70.7)
		Anxiety and depression	29 (29.3)
**Quality of life**	QLQ-H&N35 ^2^ (*n* = 101)	<P25 (46)	28 (27.7)
		P25–P75	46 (45.5)
		>P75 (60)	27 (26.7)
**Variable referring to vocal disability**	VHI ^3^ (*n* = 101)	Mild	19 (18.8)
		Moderate	58 (57.4)
		Severe	24 (23.8)

HAD-A: HAD-Anxiety; HAD-D: HAD-Depression; HAD-A&D: HAD Anxiety & Depression.^1^ Two missing scores; ^2^ The value of the P25 and P75 values is shown in parentheses; ^3^ One of the classifications has very few subjects. We recoded the variable in order to be able to make comparisons, joining in this case the patients classified as severe and grave (of these, there were only 3 patients).

**Table 3:** SECEL original and Spanish version (S-SECEL, preview version). The 35 items are shown after the process of translation and cultural adaptation.

Item	Original SECEL	Spanish Version of SECEL
**1**	Are you relaxed and comfortable around other people in speaking situations?	¿Se siente relajado y cómodo al hablar con otras personas?
**2**	Would you describe yourself as a low-keyed, calm person?	¿Se describiría como una persona tranquila y reservada?
**3**	Are you an active, “outgoing”, talkative person?	¿Se considera una persona activa, extrovertida y habladora?
**4**	Do you admit to the person you are speaking to that you had a laryngectomy?	¿Suele decirle a la gente con la que habla que está laringectomizado?
**5**	Do you think your speech improves with the amount of time you use it?	¿Cree que su habla empeora con el paso del tiempo?
**6**	Do you find that you frequent clubs, meetings, or lodges less often because of your speech?	¿Cree que han disminuido sus reuniones sociales o asistencia a eventos a consecuencia de su forma de hablar?
**7**	Do you have difficulty having getting people’s attention to speak?	¿Tiene dificultades para atraer la atención de la gente cuando habla?
**8**	Do you have difficulty yelling or calling out to people?	¿Tiene dificultad para gritar o dar voces a la gente?
**9**	Do you find that people are unable to understand you?	¿Siente que la gente tiene dificultad para entenderle cuando habla?
**10**	Do you find that people are unable to understand you?	¿Nota que tiene que repetir las cosas varias veces para que le entiendan?
**11**	Do you have trouble with speaking: in large groups of people?	¿Tiene dificultades para hablar en grupos grandes de gente?
**12**	In small groups of people?	¿Tiene dificultades para hablar en grupos pequeños de gente?
**13**	With one person?	¿Tiene dificultades para hablar con una persona?
**14**	In different rooms of your house (apartment, residence)	¿Tiene dificultades para hablar a cierta distancia de otra persona?
**15**	In loud or noisy places	¿Tiene dificultades para hablar en ambientes ruidosos?
**16**	On the telephone?	¿Tiene dificultades para hablar por teléfono?
**17**	In the car, bus or while traveling?	¿Tiene dificultades para hablar en el coche, en el autobús o viajando?
**18**	Does your speech cause you to:Have difficulty when attending parties or social gatherings?	¿Cree que su habla le provoca dificultades para acudir a fiestas o encuentros sociales?
**19**	Use the telephone less often than you would like?	¿Cree que su habla le provoca usar el teléfono menos de lo que le gustaría?
**20**	Feel left out when you are with a group of people?	¿Cree que su habla le provoca sentirse apartado cuando está con un grupo de personas?
**21**	Limit your social life or personal life?	¿Cree que su habla le provoca limitación de su vida social o personal?
**22**	Does your speech cause you to feel:Depressed?	¿Su habla le hace sentirse deprimido/a?
**23**	Frustrated when talking to family and friends and they can’t understand you?	¿Su habla le hace sentirse frustrado/a cuando habla con su familia o amigos y no lo entienden?
**24**	Different or peculiar?	¿Su habla le hace sentirse diferente o peculiar?
**25**	Do you hesitate to meet new people because of your speech?	¿Evita conocer a personas nuevas a causa de su forma de hablar?
**26**	Do you get left out of conversations because of your speech?	¿Se siente apartado de las conversaciones por su forma de hablar?
**27**	Do you avoid speaking with other people because of your speech?	¿Evita hablar con otras personas por su forma de hablar?
**28**	Do people tend to fill in words or complete sentences for you?	¿La gente tiende a completar sus frases cuando habla?
**29**	Do people interrupt you while you are speaking?	¿La gente le interrumpe mientras está hablando?
**30**	Do people tell you that they can’t understand you?	¿La gente le dice que no le entienden cuando habla?
**31**	Do the people you speak with get annoyed with you because of your speech?	¿Cree que la gente se molesta por su forma de comunicarse?
**32**	Do people avoid you because of your speech?	¿Cree que la gente le evita por su forma de hablar?
**33**	Do people speak to you differently because of your speech?	¿Nota que la gente se comunica con usted de forma diferente por su forma de hablar?
**34**	Do your family and friends fail to understand what it’s like to communicate with this type of speech?	¿Su familia o amigos no entienden sus dificultades por su forma de comunicarse?
**35**	Do you talk the same amount now as before your laryngectomy?	¿Habla lo mismo ahora que antes de la laringectomía?, ¿por qué?

**Table 4:** Dimensionality models tested using EFA and CFA of the SECEL scale in the process of adaptation to the Spanish population.

FA	Model	χ2 (df)	χ2/df	BIC/ECVI	CFI	RMSEA ^1^ [90%CI]	RMSR/SRMR	S
CFA	M0 (k = 34)	407.392 (524)	0.77	5.549	1	0.000 (0–0)	0.086	0.937
EFA ^2^	M1 (k = 21)			**481.09**	**0.994**	**0.036**	**0.075**	**0.976**
CFA	M1 (k = 21)	**93.98 (186)**	**0.50**	**1.840**	**1**	**0.000 (0–0)**	**0.065**	**0.991**
CFA	M2 (k = 21)	126.37 (180)	0.65	2.284	1	0.000 (0–0)	0.073	0.985

FA: factorial analysis type. Model M0: Request for 3 factors with k = 34 items (same model found by Blood). M1: 2-factor model, 21 items. It is the simplest model and best adjusted by EFA, and it is the model that has the best fit through the CFA. M2: 21 items, one-dimensional model. BIC/ECVI: Bayesian Information Criterion/Expected Cross Validation Index, the most satisfactory value is the lowest. CFI: Comparative Fit Index with satisfactory values if ≥0.95 and χ2/df < 3. RMSEA: Root Mean Square Error of Approximation, satisfactory reference values are RMSEA ≤ 0.06. RMSR/SRMR: Root Mean Square of Residuals in EFA and sCFA, and Standardized Root Mean Square of Residuals in CFA, satisfactory reference values are < 1/√N (0.09 in this case) ^1^: FACTOR does not provide the value of the limits of the RMSEA interval; CI: confidence interval; S: Bentler’s simplicity index with satisfactory reference values of S > 0.95; ^2^: in all the models, the KMO test index was satisfactory, showing values > 0.8; the determinant of the polychoric correlation matrix of all the models tested was < 10^−6^ and the Bartlett test was statistically significant. The values obtained in EFA and CFA for a 2-factor model (M1) are indicated in bold.

**Table 5:** Descriptive statistics of the items of the original SECEL questionnaire in the adaptation for a sample of Spanish laryngectomized patients. The factor loadings of the original model (M0) and of the model M1 (EFA and CFA) are shown once the items have been eliminated.

SECEL (Original Version)	Descriptive Statistics								Factor Loads ^2^					
									CFA M0 k = 34	EFA M1k = 21			CFA M1 k = 21	
	Items ^1^	M	SD	Asy	Kur	HIC	^F^Alpha	^I^Alpha		F1	F2	HIC	F1	F2
**G.S**	1 ^3^	1.13	0.997	0.417	−0.915	0.560	0.208	0.903	0.623					
	2 ^3^	0.93	1.05	0.720	−0.810	−0.048		0.912	−0.069					
	3 ^3^	0.97	0.964	0.402	−1.16	0.292		0.906	0.343					
	4 ^3^	1.16	1.32	0.452	−1.60	0.021		0.913	0.019					
	5 ^3^	1.31	1.14	0.200	−1.38	0.485		0.904	0.651		0.549	0.501		**0.682**
**Env.S**	6	1.21	1.19	0.357	−1.42	0.586	0.853	0.902	0.761	0.788		0.569	**0.771**	
	7	1.34	1.02	0.196	−1.07	0.509		0.903	0.580	0.622		0.513	**0.589**	
	8	2.48	0.986	−1.59	0.990	0.301		0.906	0.288					
	9	1.86	0.849	−0.130	−0.856	0.675		0.901	0.644		0.612	0.685		**0.688**
	10	2.01	0.818	−0.242	−0.928	0.629		0.902	0.567		0.738	0.621		**0.602**
	11	2.12	1.02	−0.872	−0.445	0.457		0.904	0.488					
	12	1.35	1.06	0.234	−1.16	0.697		0.900	0.835		0.633	0.709		**0.889**
	13	0.80	0.906	0.982	0.177	0.529		0.903	0.533		0.678	0.548		**0.592**
	14	2.23	0.904	−0.801	−0.516	0.452		0.904	0.451		0.575	0.470		**0.505**
	15	2.50	.0.879	−1.64	1.51	0.157		0.908	0.123					
	16	2.30	1.01	−1.11	−0.149	0.403		0.905	0.432		0.924	0.395		**0.416**
	17	1.71	1.13	−0.225	−1.35	0.499		0.903	0.603		0.784	0.508		**0.660**
	18	1.37	1.20	0.145	−1.50	0.602		0.902	0.782	0.577		0.612	**0.813**	
	19	2.22	1.08	−1.07	−0.303	0.385		0.905	0.442		0.738	0.379		**0.445**
**Att.S**	20	1.11	1.11	0.447	−1.22	0.578	0.858	0.902	0.710	0.893		0.570	**0.728**	
	21	1.23	1.17	0.301	−1.43	0.616		0.901	0.815	0.630		0.626	**0.827**	
	22	0.75	0.953	1.08	0.131	0.626		0.902	0.645	0.779		0.609	**0.635**	
	23	1.23	1.12	0.361	−1.25	0.560		0.902	0.616	0.510		0.562	**0.694**	
	24	1.01	1.12	0.725	−0.883	0.521		0.903	0.627	0.727		0.527	**0.651**	
	25	0.69	1.05	1.24	0.084	0.550		0.903	0.603	0.713		0.529	**0.609**	
	26	0.88	0.972	0.842	−0.338	0.574		0.902	0.633	0.804		0.617	**0.666**	
	27	0.88	1.09	0.857	−0.695	0.581		0.902	0.712	0.666		0.592	**0.708**	
	28	1.22	0.965	0.227	−0.968	0.462		0.904	0.499					
	29	0.74	0.833	0.729	−0.562	0.104		0.908	0.088					
	30	1.51	0.870	0.109	−0.644	0.417		0.905	0.428					
	31	0.58	0.919	*1.56*	1.44	0.372		0.905	0.382					
	32	0.48	0.844	1.76	2.17	0.441		0.904	0.394					
	33	0.92	0.945	0.668	−0.591	0.444		0.904	0.453	0.443		0.428	**0.451**	
	34	0.46	0.900	1.90	2.36	0.363		0.905	0.373					
**r**										0.636			0.702	
**CR**												0.916	0.845	

^1^ item numbering in original SECEL; ^2^ factor loading of the scales in the M0 model proposed by Blood. ^3^ inverse item. G.S, Env.S and Att.S: General, environment and attitude subscale, respectively. M, SD, Asy, Kur: Mean, Standard Deviation, Asymmetry, and Kurtosis, respectively, eliminating items with a mean close to the highest or lowest value of the scale (>2.40 or <0.60), a standard deviation (SD) < 1 [29] and skewness and/or kurtosis > 1.5 [18]. HIC: corrected homogeneity index; items with HIC < 0.25 were also eliminated. ^F^Alpha and ^I^Alpha mean Alpha of each factor and Alpha if item deleted, respectively. CFA M0: Factor loadings in the M0 Model in CFA; EFA M1: Factor loadings in the M1 Model in EFA; CFA M1 = Factor loadings in the M1 Model in CFA. k: number of items in the tested model. r: the correlation between the factors derived from the EFA and the CFA in M1; in the EFA, a 2-factor model (M1) that showed a high correlation between both factors (r = 0.636) was obtained. CR: composite reliability. In bold, the factor loads of the items selected in the CFA are highlighted. Italics indicate the data that motivated the exclusion of the item.

**Table 6:** Internal consistency reliability of S-SECEL and test–retest correlation.

	Reliability of the S-SECEL			Correlation Test–Retest
	Cronbach’s Alpha	McD ω	CR	Pearson’s
**F1**	0.884	0.882	0.916	0.697
**F2**	0.846	0.943	0.845	0.790
**T**	0.909	0.907	-	0.742

F1, F2, T: S-SECEL factors (F1: Experienced sensations (12 items), F2: Perceived limitations, (9 items) and T: Total scale). Cronbach’s Alpha: Standardized Cronbach’s Alpha, McD ω: McDonalds’ Omega ordinal. CR: composite reliability, values greater than 0.70 in the reliability tests are considered adequate. Pearson’s correlation: values of r ≥ 0.20, r ≥ 0.50 and r ≥ 0.80 express a minimal, moderate and strong correlation, respectively.

**Table 7:** Correlation between the factors of the S-SECEL scale (F1, F2 and Total) and the variables of mental health (HADS anxiety and depression), QoL (QLQ-H&N35) and perceived vocal disability (VHI).

Pearson’s Correlation							
	F1	F2	Total	Anxiety *	Depression *	VHI-30 *	QLQ-H&N35
F1	1	0.584 **	0.926 **	0.617 **	0.722 **	0.784 **	0.597 **
F2		1	0.847 **	0.381 **	0.487 **	0.528 **	0.523 **
Total			1	0.581 **	0.695 **	0.759 **	0.631 **
Anxiety *				1	0.731 **	0.669 **	0.658 **
Depression *					1	0.689 **	0.638 **
VHI-30 *						1	0.633 **
QLQ-H&N35							1

*: variables considered metric; **: *p* value < 0.001. Values of r ≥ 0.20, r ≥ 0.50 and r ≥ 0.80 express a minimal, moderate and strong correlation, respectively.

## Data Availability

The data presented in this study are under the custody of the Health Service of the Principality of Asturias and are therefore unavailable for sharing. The data are available on request from the corresponding author after an appropriate data sharing and access agreement is formally completed.
